# Veracity Judgments Based on Complications: A Training Experiment

**DOI:** 10.3390/bs14090839

**Published:** 2024-09-19

**Authors:** Haneen Deeb, Aldert Vrij, Jennifer Burkhardt, Sharon Leal, Samantha Mann

**Affiliations:** School of Psychology, Sport and Health Sciences, University of Portsmouth, Portsmouth PO1 2DY, UK; aldert.vrij@port.ac.uk (A.V.); jennifer.burkhardt@port.ac.uk (J.B.); sharon.leal@port.ac.uk (S.L.); samantha.mann@port.ac.uk (S.M.)

**Keywords:** lie detection, veracity judgment, complications, verbal cues, interviews

## Abstract

Research has shown that complications are more common in truth tellers’ accounts than in lie tellers’ accounts, but there is currently no experiment that has examined the accuracy of observers’ veracity judgments when looking at complications. A total of 87 participants were asked to judge 10 transcripts (five truthful and five false) derived from a set of 59 transcripts generated in a previous experiment by Deeb et al. Approximately half of the participants were trained to detect complications (Trained), and the other half did not receive training (Untrained). Trained participants were more likely to look for complications, but they did not detect them accurately, and thus their veracity judgments did not improve beyond Untrained participants’ judgments. We discuss that the training may have been too brief or not sensitive enough to enhance decision-making.

## 1. Introduction

Deception researchers have strived to elicit differences between truth tellers and lie tellers through verbal [1,2], non-verbal [3], and physiological [4] means. In the present experiment, we are interested in verbal cues that have shown promising results. Unlike physiological and some non-verbal behaviour indicators that require resources such as connecting suspects to body sensors [5] or making them wear suits [6], verbal cues can be easily examined in forensic settings. Investigators can also detect verbal differences between truth tellers and lie tellers in real time.

A verbal cue that has recently gained attention is “complications”. Examples of complications include someone who (a) could not get the air conditioning to work in the hotel room, (b) was not able to reach a certain venue due to a blocked road, or (c) forgot to properly tie their shoes before running. A meta-analysis [7] has shown that complications are more common in truthful accounts than in false accounts; see also [8,9]. However, there is no experiment to date that has examined if observers can accurately identify complications following a brief training and if training observers to use complications as a veracity cue enhances judgment accuracy. In the present experiment, we put those two questions to the test.

### 1.1. Complications as a Verbal Veracity Cue

Veracity cues are indicators that distinguish truthful and false accounts [10]. Cues that can be measured without equipment can be verbal (based on speech content such as the number of details in an account) or non-verbal (based on body language such as gaze aversion). Over the few past decades, it has been established that verbal veracity cues are more diagnostic than non-verbal veracity cues [11,12]. Thus, the focus of recent deception research has been on verbal veracity cues, e.g., [13,14].

One of the most researched verbal veracity cues is total details (the total amount of informative and unique details in an account), which has demonstrated promising diagnosticity [15,16]. However, there are limitations in using total details as a veracity cue. First, total details can vary substantially within truthful and false accounts due to individual differences [17]. For example, a talkative person may provide a more detailed account about the same witnessed event than another person who is less talkative, even if both persons are truthful. Second, total details cannot be easily counted in real time [18,19]. Counting total details requires transcribing the interviews and then manually counting the number of details within the transcripts (note that total details differ from word count, so counting total details cannot be performed using computerized software). In forensic settings such as intelligence, informant or security interviews, these resources are often lacking. It is therefore more practical for investigators to look instead at verbal cues that can be assessed while the interview is taking place. Third, total details may not be as diagnostic as originally thought due to methodological issues [20]. For instance, the studies may have had a low power to detect true effects; the authors may have selectively reported significant results compared to non-significant results; and/or there may have been a publication bias. Fourth, the total details cue is vulnerable to countermeasures. If investigators only consider the number of details someone provides when making veracity judgements without considering the content (type of detail), lie tellers just have to talk to make a sincere impression.

Given these limitations, deception researchers recommended looking for other verbal cues than total details, specifically for different types of details [21,22,23]. Complications are a type of detail that appears to be diagnostic [7]. Complications are one of the verbal criteria (labelled as “unexpected complications”) of the Criteria-Based Content Analysis (CBCA), the most researched lie detection tool to date. CBCA comprises a checklist of 19 verbal criteria that are indicative of truth telling and are thus more likely to occur in truthful accounts than in false accounts [24,25]. Unexpected complications in CBCA are defined as “Description of unexpected difficulties that interrupted the normal progress of the event” [15,26]. However, in the present experiment, we do not use CBCA’s definition of complications. Instead, we use the definition introduced by Vrij et al. [27]: “A complication is an occurrence that makes a situation more difficult than necessary”. According to this definition, complications are not necessarily unexpected, and they do not have to interrupt the normal progress of events, but they make the event less smooth than if the occurrence had not happened. Take, for example, someone who reports that they passed by a shop to buy lunch and then went to work. If they mention that they accidently dropped their lunch and had to buy lunch again, they include two complications (dropping lunch and buying lunch again), according to both the CBCA and Vrij et al.’s definitions, because the events are unexpected and interrupt the normal progress of events. If the person adds that when buying their lunch for the second time, they had to queue in the shop, this is counted as a complication only according to Vrij et al.’s definition but not according to the CBCA criteria. People are often expected to queue in shops (hence, this is not a complication, according to CBCA), but the queuing aspect made the event more difficult for the person than if there had not been a queue (hence, this is a complication, according to Vrij et al. [27]’s definition).

Complications, when using Vrij et al.’s definition, show promising results as a verbal veracity cue [7] and replicates the pattern of findings for CBCA’s unexpected complications criterion [15,28]. Truth tellers’ episodic memories of events are likely to contain complications [29,30]. In contrast, lie tellers are unlikely to include complications in their narratives because their preferred strategy in interviews is to keep their accounts simple [31,32]. In addition, lie tellers think that complications sound suspicious, so they are less willing than truth tellers to report them [33].

Lie tellers often employ countermeasures in interviews to come across as honest [34,35]. Complications seem, to some extent, resistant to lie tellers’ countermeasures, as demonstrated by two experiments [36,37]. Lie tellers who were informed prior to being interviewed that truth tellers generally provide more complications than lie tellers still provided fewer complications than truth tellers during the interview.

### 1.2. The Present Experiment

There is yet no lie detection experiment to inform us on whether decision-making is enhanced when observers make veracity judgments based on complications. A meta-analysis [38] has shown that informing observers of which (diagnostic) cues to look for (e.g., contextual details) enhances the accuracy of veracity judgments. We aimed to understand if judgment accuracy is also enhanced when observers look for complications as a veracity cue. Approximately half of the participants received training in complications. They were presented with background information and examples about complications. They were then asked to read truthful and false transcripts, to judge their veracity, and to highlight complications and other details that they believed helped them with making their judgments. Untrained participants did not receive any instructions on complications and were only asked to judge the veracity of transcripts and to highlight details that they believed were helpful when making judgments.

People generally show a truth bias when making veracity judgments as they judge more accounts as truthful than as deceptive [39]. We thus expected our sample to also demonstrate a truth bias. We further predicted that training participants to identify a diagnostic cue (complications) will make them more attentive to this cue and hence more likely to judge transcripts with complications as truthful and transcripts that lack complications as false, which should, in turn, enhance judgment accuracy for both truthful and false transcripts. In contrast, participants in the Untrained condition will judge transcripts at chance levels, as demonstrated in previous meta-analyses e.g., [40].

### 1.3. Hypotheses

The hypotheses are pre-registered in the repository of the Open Science Framework at https://osf.io/d69ve/. We have changed the hypotheses following a reviewer’s comment: The second (truthful transcripts will be judged more accurately than false transcripts) and fourth (interaction) pre-registered hypotheses were deleted to avoid redundancy (truth bias will, by default, yield higher judgment accuracy for truthful than for false accounts, and a higher judgment accuracy among Trained participants can coexist with truth bias to a certain extent).

**Hypothesis** **1** : *Participants in the Trained condition will make more accurate judgments than participants in the Untrained condition.*

**Hypothesis** **2** : *Participants will show a truth bias, rating significantly more transcripts as truthful.*

**Hypothesis** **3** : *The number of highlighted complications (Hypothesis 3a) and accurately highlighted complications (Hypothesis 3b) will be higher in the Trained condition than in the Untrained condition.*

## 2. Materials and Methods

### 2.1. Participants

The sample size was determined using G*Power 3.1.9.7 software. The power analysis revealed that to obtain 95% power, a medium effect size of *f* = 0.25 (the smallest effect of interest in applied deception research) and α = 0.05 were required, and at least 54 participants should be recruited.

The experiment was advertised to students and staff members at the University and to members of the public. Participants received GBP 15 as remuneration. A total of 88 participants took part. However, one participant judged only two transcripts (missing data), so their data were not included in the analysis. The final sample comprised 87 participants (*M*_Age_ = 26.25 years; *SD*_Age_ = 7.89), of which 55 were females, 31 were males, and 1 described themselves as other.

### 2.2. Design

We carried out a 2 (Training: Trained, Untrained) × 2 (Veracity: truth, lie) mixed design with Training as the between-subjects factor and Veracity as the within-subjects factor. Thirty-eight participants were assigned to the Trained condition and 49 to the Untrained condition. The differences in cell sizes were the result of the research assistant recruiting a different number of participants for each condition. However, we do not expect this to have affected the statistical power given that the minimum number of participants needed in each Training condition was 27. For the analyses, we ran *t*-tests and analyses of variance, for which unequal cell sizes are not problematic. The dependent variables were sensitivity, response bias, number of highlighted complications, and the complications hit rate.

### 2.3. Stimuli

The transcripts were derived from the experiment by Deeb et al. [1]. In the original experiment, participants (*N* = 243) reported in three interviews—each one week apart—about a memorable event (e.g., traveling abroad, attending a concert) that they truly or falsely experienced. Lie tellers were matched to an event that truth tellers reported (e.g., if a truth teller reported about a trip to Italy, a lie teller was asked to report a similar event). In the first two interviews, participants were or were not subjected to a Model Statement, which is an audiotape of a person providing an example of a detailed free recall of an event that is irrelevant to the event under investigation [41]. In the third interview, all participants were asked for a free recall. Only responses (transcripts) from the Model Statement-absent condition in the first interview (*n* = 59) were used in the present experiment to eliminate the effects of delay and interview technique.

We asked participants to rate 10 transcripts (five truthful and five false) from the pool of 59 transcripts (29 truthful and 30 false). Thus, participants made 870 judgments in total. The 10 transcripts were randomly presented such that each of the 59 transcripts was judged an approximately equal number of times by participants (~15 times). Each transcript was presented to at least 12 participants and to at most 19 participants.

### 2.4. Procedure

The experiment was conducted online via Zoom. The relevant materials are available at https://osf.io/89r3a/.

For the Trained condition, the experimenter first sent participants a Google Docs link, which included the definition, background information, examples, and scientific findings concerning complications. Trained participants then underwent a practice session in which they read 12 transcripts and highlighted phrases that they thought indicated complications. They could see the answer key for each transcript on the following page. The transcripts included one or more complications and varied in length. At the end of the Google Doc, participants were presented with a practice test to assess their understanding of complications. They did not see the answer key for the practice test. We assessed if participants accurately identified complications in the practice test. We calculated a complications hit rate by dividing the total number of accurately highlighted complications by the total number of actual complications (six actual complications) in the practice test. We then ran a one sample Wilcoxon *t*-test with the complications hit rate as the dependent variable. The hit rate (*M* = 0.67, *SD* = 0.28, 95% CI [0.58, 0.76]) differed significantly from chance levels (*V* = 666, *p* < 0.001, BF_10_ = 7.216 × 10^13^), showing that Trained participants could accurately identify the majority of complications.

After Trained participants completed the practice test, the experimenter sent them another Google Docs link, through which they read the following instructions:

“You will now be asked to read transcripts (written scripts) of interviews with different individuals who discussed a memorable event they have experienced. After each transcript, you will be asked to judge whether the transcript is truthful or false. You may take as long as you need to make your decision.

The transcripts are randomly generated so they can all be truthful or false or they can be a mix of truthful and false transcripts. Thus, please refrain from making any assumptions about veracity based on the number of transcripts and base your judgments on the content of the transcripts only.

Each transcript is divided into several paragraphs to make it easier to read. The transcripts may include spelling and/or grammatical errors. Also, you will see that some may include words that were inaudible. The reason for this is that the interviews were audio-recorded and then transcribed verbatim by a human transcriber. As a consequence, it might not be as easy to read as a news article or a book.

If you can accurately detect more than 50% of the transcripts, you will be entered in a draw to win one of three prizes (£50, £75, £100). Therefore, read each transcript carefully and try your best.

While you are reading each transcript, please highlight in yellow colour any phrase or sentence that you think makes a complication. A transcript may include zero, one, or more complications.

If you highlight two words or separate parts in one phrase or sentence, we will consider those to be two (or more) different complications. Thus, if you think the phrase or sentence as a whole rather than separate parts of it is one complication, you would need to highlight all of it at a time rather than separate parts of it. If instead, you think the different parts constitute two (or more) complications, then please highlight each of them separately.

We would also like to know if you use other details for judging whether a transcript is truthful or false. This will enable us to understand why you think the transcripts are truthful or false.

Therefore, we ask you to also highlight in green colour any details other than complications if you think they help you in making your judgement about whether the transcript is truthful or false. The details may be one or more words, phrases, or sentences within the transcript.

Again, if you highlight two words or separate parts in one phrase or sentence, we will consider those to be separate details that helped you with your decision. Thus, if you think the phrase or sentence as a whole rather than separate parts of it helped with your decision, please highlight all of it at a time rather than separate parts of it.

In sum, while you are reading each transcript, please highlight any complications (in yellow colour) and/or other details (in green colour) you rely on for making your judgement.

Let the experimenter know if you have any questions.”

Participants were then directed to the transcripts, each on a separate page. At the top of each transcript, Trained participants were reminded as follows: “Please highlight any complications (in yellow) and/or other details (in green) that help you in deciding whether the transcript is truthful or false.”

At the bottom of each transcript, all participants made dichotomous veracity judgments (truthful/false). Transcripts were randomly presented, and each participant judged five truthful and five false transcripts. At no stage during the experiment were participants informed about the total number of transcripts they would view.

Participants in the Untrained condition did not receive any training or practice test, but they were immediately asked to judge the transcripts and to highlight any details that helped them with reaching their decisions. They received similar instructions to those in the Trained condition, excluding the instructions concerning complications.

After completing the judgments task, all participants were sent a Qualtrics link to the end-of-study questionnaire. They provided their demographics and were asked an open question on what cues they relied on when making their judgments. Participants also rated on seven-point scales (1 = not at all to 7 = to a great extent) if they felt confident in their judgment accuracy; if the highlighting task (a) was distracting, (b) was difficult, and (c) required mental effort; if they felt rushed; if they felt anxious; and if there was any kind of distractor around while they were judging the transcripts.

Participants in the Trained condition also rated on seven-point scales (1 = not at all to 7 = to a great extent) the extent to which they (a) looked for complications while making their judgments; (b) believed that looking for complications enhanced their judgment accuracy; (c) found it difficult to look for complications in the transcripts; and (d) understood what complications mean. At the end of the experiment, all participants read the debrief form and received their remuneration.

### 2.5. Coding

The first author coded data from all participants, and another research assistant coded data from 15 participants (17% of the total number of participants). The coders were kept blind to the veracity status of the transcripts (in the original and present study). The coders first counted the total number of highlighted details (green) and/or complications (yellow) in the transcripts. Inter-rater reliability was measured via the Intra-Class Correlation Coefficient. Reliability was perfect (ICC = 1.00) for the total number of highlighted details in the Trained and Untrained conditions and for the total number of highlighted complications in the Trained condition.

To count complications, the coders compared the coded complications in the original transcripts [1] with the highlighted complications by participants in the present experiment. For the Trained condition, the coders classified the yellow highlighted text as a hit (actual complication), a false alarm (highlighted text is not a complication), or a miss (actual complication not highlighted). Inter-rater reliability was near perfect for each of the three variables (ICC = 0.99). For the Untrained condition, the coders counted the number of highlighted details (green) that were actually a complication (ICC = 1.00).

We also coded the open responses in the end-of-study questionnaire for the question on the cues that participants relied on when making their judgments. Inductive thematic coding was employed. The first author generated thematic cues based on participants’ responses by grouping similar responses together under a single theme. When the same response could fit under more than one theme, it was allocated to each of those themes. A research assistant served as the second coder and grouped all the responses based on the corresponding themes generated by the first author. The assistant first received definitions and examples of each theme and then coded a few responses. Discrepancies between coders were discussed, and the assistant coded the remaining responses independently. Inter-rater reliability was excellent (Cohen’s κ = 0.88). Discrepancies were then resolved through a discussion between the coders.

## 3. Results

Based on recommended practice [42,43], we ran frequentist and Bayesian analyses to test the data. JASP 0.18.3 statistical software was used for the analyses. For the frequentist analyses, we ran *t*-tests and univariate analyses of variance as appropriate to compare means. We used non-parametric tests where statistical test assumptions were not met, and we reported the corresponding *t*-statistic or *F*-statistic used. We corrected for multiple testing by dividing the conventional *p*-value of 0.05 by the number of hypotheses (0.05/4), and thus our significance cut-off score was 0.0125.

For the Bayesian analyses, we used BF_10_ values. BF_10_ values above three indicate stronger evidence for the alternative hypothesis over the null hypothesis, values close to one indicate inconclusive evidence, and values below 0.33 indicate stronger evidence for the null hypothesis [44]. We also calculated Cohen’s *d* to measure the effect size, i.e., the extent to which the effect is practically meaningful. Cohen’s *d* coefficients below 0.5 are small, coefficients between 0.5 and 0.8 are medium, and coefficients above 0.8 are large [45]. If the confidence interval crosses zero, that means that the magnitude of the effect is not meaningful [46].

Scholars have recommended the use of signal detection theory to measure sensitivity (discriminability) and response bias in experiments in which observers discriminate between different stimuli [47,48]. Sensitivity tests the performance of observers (whether they can accurately discriminate between stimuli [truthful and false transcripts in the present experiment]). Response bias tests for general systematic tendencies among observers when judging stimuli. We use these two measures to test Hypotheses 1 and 2.

### 3.1. Sensitivity

We calculated d-prime (*d’*) to test sensitivity or accuracy performance. The formula for *d’* is z(hit rate)—z (false alarm rate). Values close to zero indicate that the participants could not accurately discriminate between truthful and false transcripts, and values above zero indicate increased discriminability. A one-sample Student *t*-test with *d’* as the dependent variable showed that the *d’* score did not differ significantly from zero, *t*(86) = 0.00, *p* = 1.00, *M* = 0.00, *SD* = 1.38, BF_10_ = 0.12, which means that participants were generally not good at discriminating truthful transcripts from false transcripts.

An independent-samples Student *t*-test with Training (Trained, Untrained) as the factor and *d’* as the dependent variable revealed that the Trained and Untrained conditions did not significantly differ, *t*(85) = −0.09, *p* = 0.930, BF_10_ = 0.23, and the effect size was negligent (see Table 1). Hence, both Training conditions showed low sensitivity and could not differentiate truthful and false transcripts. Hypothesis 1 was thus not supported.

**Figure 1:**
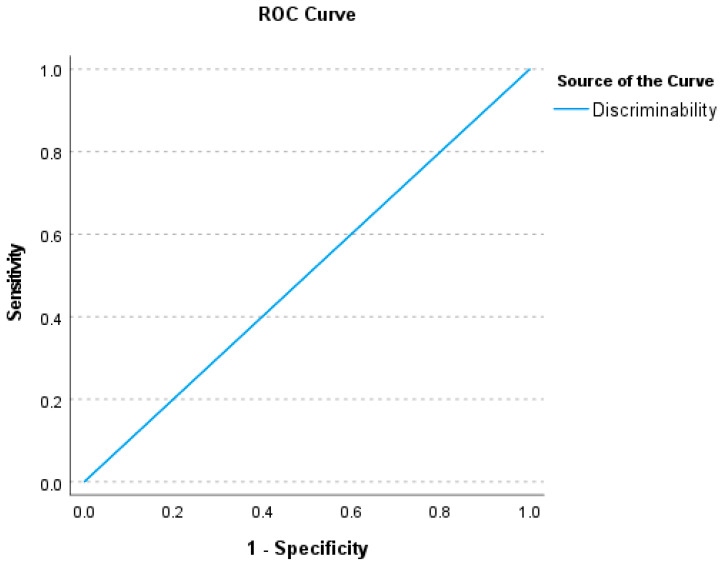
ROC curve for participants’ veracity judgments.

We also display in Table 2 the means of hits (false transcripts judged as false), false alarms (truthful transcripts judged as false), misses (false transcripts judged as truthful), and correct rejections (truthful transcripts judged as truthful), which did not seem to differ from each other. We also created a receiver operating characteristic (ROC) curve based on the hits and false alarms in the data (see Figure 1). Discriminability in the ROC curve is measured using the area under the curve (AUC) score. AUC scores around 0.5 suggest chance discriminability and scores between 0.7 and 1 suggest enhanced discriminability. The AUC score was 0.501, which confirmed that training did not enhance discriminability.

### 3.2. Response Bias

To calculate the response bias criterion (*c*), we used the following formula: −0.5*(z [hit rate] + z [false alarm rate]). Values close to zero indicate no bias, values below zero suggest a lie bias (tendency to judge transcripts as false), and values above zero signal a truth bias (tendency to judge transcripts as truthful).

A one-sample Wilcoxon *t*-test with *c* as the dependent variable showed that *c* did not differ significantly from zero, *M* = 0.00, *SD* = 0.73, *V* = 1784, *p* = 0.583, BF_10_ = 0.14. An independent-samples Welch *t*-test with Training as the factor and *c* as the dependent variable revealed that the Untrained and Trained conditions did not significantly differ in response bias, and the effect size was small (see Table 1 for the means and statistical output). Hence, there was no bias in the data, and Hypothesis 2 was not supported.

### 3.3. Highlighting Task

#### 3.3.1. Total Number of Highlighted Complications

In Hypothesis 3a, we predicted that participants in the Trained condition would be more likely than those in the Untrained condition to highlight complications in the transcripts. To test this hypothesis, we examined all the highlighted complications in the Trained condition and the highlighted details in the Untrained condition that were actually a complication (labelled as the total highlighted complications). We then ran repeated-measures univariate analysis of variance (ANOVA) with Veracity (truth, lie) as the within-subjects factor, Training (Trained, Untrained) as the between-subjects factor, and total highlighted complications as the dependent variable. Geisser–Greenhouse *F*-statistics were used to correct for violations of assumptions. Significant effects emerged for the Training and Veracity main effects (see Table 1 and Table 3) and for the Veracity × Training interaction effect, *F*(1, 85) = 9.81, *p* = 0.002, BF_10_ = 16.55. Participants in the Trained condition highlighted more complications than those in the Untrained condition, and this effect was large (see Table 1), which supported Hypothesis 3a. Also, participants highlighted more complications in the truthful transcripts than in the false transcripts (see Table 3). Table 4 shows that the Veracity effects were true and of medium size in the Trained and Untrained conditions, but the evidence and effects were stronger in the Trained condition (BF_10_ = 45.09, *d* = 0.71) than in the Untrained condition (BF_10_ = 28.67, *d* = 0.53).

#### 3.3.2. Complications Discriminability

Hypothesis 3b predicted that participants in the Trained condition would be more likely than those in the Untrained condition to highlight complications accurately. To test this hypothesis, we examined the accurately highlighted complications in the Trained condition and the highlighted details in the Untrained condition that were actually a complication (labelled as hits). Next, we computed the complications hit rate (total number of hits in the Trained or Untrained condition/Total number of actual complications in the transcript). We then ran a repeated-measures ANOVA with Veracity (truth, lie) as the within-subjects factor, Training (Trained, Untrained) as the between-subjects factor, and the complications hit rate as the dependent variable. Geisser–Greenhouse *F*-statistics were used to correct for violations of assumptions. The Training and Veracity main effects (see Table 1 and Table 3) and the Veracity × Training interaction effect, *F*(1, 85) = 1.98, *p* = 0.163, BF_10_ = 0.54, were not significant and received weak evidence, so Hypothesis 3b was not supported.

### 3.4. End-of-Study Questionnaire

We ran a series of independent-samples *t*-tests on the rated items (all seven-point Likert scales). The results are reported in Table 5. The ratings were generally similar for participants in the Trained and Untrained conditions except that those in the Trained condition were significantly and moderately more likely to self-report that the highlighting task required mental effort.

We also ran one-sample *t*-tests on items pertinent to the Trained condition only. As shown in Table 5, participants in the Trained condition self-reported—significantly above chance levels—that they looked for complications while making their judgments, they believed that looking for complications enhanced their judgment accuracy, and they understood what complications meant, although they found looking for complications a difficult task.

Participants were asked an open question on what cues they relied on when making their judgments. The self-reported cues can be found in Table 6. For each cue, we calculated a percentage score (frequency of reported cue/total number of Trained or Untrained participants). Participants in the Trained condition were more likely than those in the Untrained condition to report looking for detailedness (“the amount of extra details in the story”), complications, over-explanations (“When the individual made too many points about a subject, I thought they were lying”), and the tone of the transcript (“I looked at how people spoke”).

Participants in the Untrained condition were more likely to report looking for logical/common knowledge details (“I looked at whether they used general or personal knowledge”), language (“rephrasing of certain sentences”), keywords or specific details (“I looked at specificity and tried to find key words”), consistency (“if the speaker was repeating words”), emotions (“personal feelings”), verbatim accounts (“People describing situations along with the exact dialogues experience it in real”), and pauses (“stopping midway while explaining”).

Participants in the Trained and Untrained conditions did not differ in the extent to which they looked at hesitations (“when they seemed unsure or unconfident”) and inconsistency (“if they seemed to contradict themselves”). Some participants reported other cues that were not frequently mentioned (“using instinct”; “reporting embarrassing details”), so these were categorised under the “other” theme.

## 4. Discussion

We tested if participants who are trained to detect complications and to use them as cues when making veracity judgments would show enhanced performance in identifying complications and truth/lie detection accuracy compared to Untrained participants. Contrary to our predictions, training participants to detect complications did not enhance judgment accuracy compared to the Untrained condition. Although the results from the practice test suggested that Trained participants could accurately identify complications to some extent (67% accuracy), the results from the main test were much less promising. Trained participants highlighted more complications than Untrained participants, but Trained participants accurately identified complications only 29% of the time (Table 1) for the truthful and false transcripts. Table 2 also shows that the misses (35.97) were higher than the hits (14.37). It thus seems that Trained participants missed accurate complications and tended to highlight details that were not complications, which suggests that participants may have misunderstood the concept of complications. It is possible that the training was too brief and/or not sensitive enough to spot complications. From experience, we know that it is challenging to understand and code complications. Research assistants who helped us code complications in previous experiments underwent more than one training session. Perhaps participants needed more practice and more interactive feedback before embarking on the main test. Moreover, previous experiments have shown that lie tellers who have learned about complications still included fewer complications in their accounts than truth tellers [36,37]. However, lie tellers underwent only one learning session in those experiments. The overall findings thus suggest that it may be difficult for people to understand the concept of complications following a brief training.

The ratings in the end-of-study questionnaire do not align with the findings from the main analyses. Trained participants reported that they looked for complications and understood what complications meant and that this cue enhanced their judgment accuracy. These findings demonstrate that participants may have thought they understood the concept of complications when they actually did not. Previous research has shown that people do not accurately reflect on their metacognitive processes in self-reports [49]. It is also probable that participants wanted to demonstrate social desirability—the tendency to present themselves positively [50]—and thus reported that they understood the concept when they knew they did not.

Trained participants also believed that looking for complications was difficult, and they were more likely than Untrained participants to self-report that the highlighting task required mental effort. The means for “mental effort” were above the midpoint for both training conditions, which implicated that the highlighting task was mentally taxing for all participants. The added cognitive load of looking for and highlighting complications in the Trained condition seemed to increase this mental effort to a greater extent, which could have ultimately affected their judgments. Indeed, previous research has shown that increased cognitive load compromises decision-making [51].

Participants could detect truthful transcripts more accurately than false transcripts, in line with our predictions. Our hypothesis was based on truth bias as proposed by the truth default theory [39]. According to this theory, people default to believing others, so they tend to judge them as truthful (truth bias), which should, in turn, increase opportunities for detecting truthful accounts accurately. However, we did not find any bias in our data. It must thus be something other than response bias that was driving the higher accuracy rate for truthful transcripts.

Street [52] proposed an alternative framework to the truth default theory. According to his Adaptive Lie Detector (ALIED) proposition, people adapt to the situation when making judgments by relying on diagnostic cues and/or on the context. If people have access to diagnostic cues, they will not display a response bias, which is a contextual factor. When diagnostic cues are unavailable, people would rely on the context. The self-reported data (Table 6) show that participants looked at several cues they thought were diagnostic, such as detailedness, logical details, complications, and consistency.

We expected that at least for the Trained condition, the lack of the truthfulness cue “complications” in transcripts would be associated with lie telling, but we did not find such an effect. Previous research has shown that our brains can process present signals more than absent signals [53]. Thus, to detect false accounts, it may be easier for observers to look for the presence of deceptiveness cues (i.e., cues present in false accounts but absent in truthful accounts) than for the absence of truthfulness cues [31]. Future research can examine how guiding observers to look at diagnostic truthfulness and deceptiveness cues can enhance judgments in both truthful and false transcripts. This aligns with the call by scholars to test more deceptiveness cues, given that (a) these cues are meagre in verbal deception research [23] and (b) the use of a combination of truthfulness and deceptiveness cues should enhance decision-making [54]. This is not to say that verbal cues should be the only cues that investigators rely on when making veracity judgments. Instead, investigators should rely on multiple sources when making judgments, including physical evidence, eyewitness evidence, and multiple veracity cues.

### Limitations and Future Directions

The training in the present experiment was brief, occurring over one session and with only limited practice. Identifying the cue “complications” likely requires more extensive training to help with enhancing veracity judgments. Future research can examine if longer training that occurs over several sessions can enhance identifying complications accurately and truth/lie detection. An interactive component can also be added to the training, such that each participant receives individualised feedback on their performance.

The present experiment was conducted online, which may have affected the training. It is probably easier to grasp whether trainees understand the to-be-learned concepts in face-to-face interactions than in an online environment. An experiment can be conducted in which face-to-face and online training in complications are compared for differences in the identification of complications and truth/lie detection.

The present experiment involved transcripts from short interviews. In real life, the interviews are usually longer and occur over several sessions. Thus, future research can examine how extensive training interacts with short versus longer interviews. Relatedly, we still do not know if lie tellers are able to include more complications in longer interviews if they prepare themselves for interviews, or if they are familiar with (a) the legal system, (b) research on how truth tellers often respond in interviews, and/or (c) cues that interviewers look for in an interview. In such cases, it can be argued that complications cease to be resistant to countermeasures, see [36,37]. However, we believe this is unlikely to occur as multiple research publications have shown that lie tellers prefer to keep their accounts simple [32,55]. We also doubt that lie tellers will realise that interviewers may look for complications. When searching the internet for “cues to deceit”, this verbal cue—and most other diagnostic verbal cues—do not emerge as veracity indicators. Further, even if lie tellers manage to report more complications with longer interviews, we would expect the same to occur for truth tellers, and so the differences in complications in truth tellers’ and lie tellers’ accounts will continue to be significant.

## 5. Conclusions

Complications have been shown to be diagnostic cues that are advantageous in practical settings, as they can be assessed in real time [7]. The overall findings of the present experiment showed that a brief training in identifying complications is not enough for effective decision-making. We thus recommend that more research is dedicated to examining whether more extensive training will benefit observers.

## Figures and Tables

**Table 1:** Descriptive and inferential statistics for veracity judgements and complications as a function of training.

	Untrained M, SD [95% CI]	Trained M, SD [95% CI]	Statistics	*p*	BF_10_	Cohen’s *d * [95% CI]
Sensitivity	−0.01, 1.26 [−0.37, 0.35]	0.02, 1.53 [−0.49, 0.52]	−0.09	0.930	0.23	−0.02 [−0.44, 0.41]
Response bias	−0.04, 0.84 [−0.28, 0.20]	0.05, 0.56 [−0.13, 0.24]	−0.64	0.521	0.27	−0.14 [−0.56, 0.29]
Total highlighted complications	9.12, 7.27 [7.04, 11.21]	39.26, 27.73 [30.15, 48.38]	53.21	<0.001	1.572 × 10^7^	−1.58 [−2.06, −1.09]
Average complications hit rate	0.21, 0.14 [0.17, 0.25]	0.29, 0.18 [0.23, 0.35]	5.48	0.022	2.48	−0.51 [−0.94, −0.07]

Note. The average complications hit rate is calculated based on the average hit rate for truthful and false transcripts.

**Table 2:** Descriptive statistics of signal detection theory outcomes for veracity judgments and complications as a function of training.

	Untrained M, SD [95% CI]	Trained M, SD [95% CI]
*Veracity Judgments*		
Hits	2.35, 1.36 [1.96, 2.74]	2.24, 1.28 [1.82, 2.66]
False alarms	2.10, 1.25 [1.74, 2.46]	1.97, 1.08 [1.62, 2.33]
Misses	2.65, 1.36 [2.26, 3.04]	2.76, 1.28 [2.34, 3.19]
Correct rejections	2.90, 1.25 [2.54, 3.26]	3.03, 1.08 [2.67, 3.38]
*Complications*		
Hits	9.12, 7.27 [7.04, 11.21]	14.37, 9.16 [11.36, 17.38]
False alarms		24.74, 21.78 [17.58, 31.89]
Misses: Complications that are not coded	27.65, 21.45 [21.49, 33.81]	32.79, 15.54 [27.68, 37.90]
Misses: Complications that are coded as details		3.18, 3.43 [2.06, 4.31]

Note. In the Untrained condition, hits are the complications that are judged as details by participants.

**Table 3:** Descriptive and inferential statistics for complications as a function of veracity.

	Truth M, SD [95% CI]	Lie M, SD [95% CI]	*F*	*p*	BF_10_	Cohen’s *d* [95% CI]
Total highlighted complications	14.61, 19.09 [10.54, 18.68]	7.70, 8.03 [5.99, 9.41]	19.96	<0.001	132.32	0.47 [0.17, 0.77]
Complications hit rate	0.24, 0.17 [0.20, 0.27]	0.26, 0.21 [0.21, 0.30]	0.58	0.450	0.23	−0.10 [−0.40, 0.19]

**Table 4:** Descriptive and inferential statistics for complications as a function of veracity and training.

	Truth M, SD [95% CI]	Lie M, SD [95% CI]	*F*	*p*	BF_10_	Cohen’s *d* [95% CI]
*Trained*						
Total highlighted complications	26.08, 23.84 [18.24, 33.91]	13.18, 9.26 [10.14, 16.23]	11.72	0.002	45.09	0.71 [0.24, 1.18]
Complications hit rate	0.30, 0.17 [0.24, 0.35]	0.28, 0.26 [0.20, 0.37]	0.12	0.731	0.25	0.09 [−0.36, 0.55]
*Untrained*						
Total highlighted complications	5.71, 5.45 [4.15, 7.28]	3.45, 2.68 [2.68, 4.22]	11.98	0.001	28.67	0.53 [0.12, 0.93]
Complications hit rate	0.19, 0.15 [0.14, 0.23]	0.23, 0.17 [0.19, 0.28]	4.65	0.036	1.34	−0.25 [−0.65, 0.15]

**Table 5:** Descriptive and inferential statistics for the rated items in the end-of-study questionnaire as a function of training.

Questionnaire Item	Untrained		Trained		*t*	*p*	BF_10_	Cohen’s *d* [95% CI]
	M (SD)	95% CI	M (SD)	95% CI				
To what extent do you feel confident you judged the transcripts accurately?	4.41 (1.26)	4.05, 4.77	4.21 (1.36)	3.76, 4.66	0.70	0.489	0.26	0.15 [−0.27, 0.58]
The highlighting task:								
Was distracting	2.67 (1.75)	2.17, 3.18	3.16 (1.97)	2.51, 3.80	−1.20	0.236	0.81	−0.26 [−0.69, 0.16]
Was difficult	3.90 (1.62)	3.43, 4.36	4.24 (1.28)	3.82, 4.66	−1.09	0.280	0.34	−0.23 [−0.65, 0.20]
Required mental effort	4.69 (1.72)	4.20, 5.19	5.74 (1.18)	5.35, 6.12	−3.35	0.001	4.26	−0.69 [−1.13, −0.25]
Did you feel rushed when judging the transcripts?	1.84 (1.20)	1.49, 2.18	2.16 (1.67)	1.61, 2.71	−1.00	0.320	0.47	−0.23 [−0.65, 0.20]
Did you feel any sort of anxiety while judging the transcripts?	2.39 (1.50)	1.96, 2.82	2.79 (1.86)	2.18, 3.40	−1.09	0.282	2.53	−0.24 [−0.67, 0.19]
Was there any kind of distractor around while you were judging the transcripts?	1.33 (0.85)	1.08, 1.57	1.92 (1.28)	1.50, 2.34	−2.47	0.016	0.62	−0.56 [−0.99, −0.13]
To what extent did you look at complications when making your judgments?			4.89 (1.53)	4.37, 5.41	19.22	<0.001	2.121 × 10^16^	
To what extent do you think that looking at complications enhanced your judgment accuracy?			4.72 (1.30)	4.28, 5.16	21.78	<0.001	6.230 × 10^19^	
To what extent did you find it difficult to look for complications in the transcripts?			4.44 (1.30)	4.01, 4.88	20.56	<0.001	6.920 × 10^17^	
To what extent do you understand what complications mean?			5.08 (1.05)	4.73, 5.44	28.99	<0.001	1.602 × 10^22^	

**Table 6:** Percentage of self-reported cues that participants in each training condition used when making judgments.

Cues	Trained	Untrained
Detailedness	55%	41%
Logical/Common knowledge details	24%	37%
Language	13%	33%
Keywords or specific details	13%	29%
Hesitation	21%	20%
Consistency	3%	20%
Emotions	3%	16%
Complications	21%	0%
Over-explanations	16%	4%
Verbatim account	3%	12%
Inconsistency	13%	10%
Tone	13%	4%
Pauses	3%	10%
Other	5%	8%

## Data Availability

The experiment is pre-registered at https://osf.io/d69ve. The data and relevant material are available in the Open Science Framework repository at https://osf.io/89r3a/.
